# Artificial Intelligence-Enabled Electrocardiography for Prediction of Sudden Cardiac Death and Malignant Ventricular Arrhythmias: A Scoping Review

**DOI:** 10.3390/jcdd13050206

**Published:** 2026-05-12

**Authors:** Ziga Mrak, Franjo Husam Naji, Dejan Dinevski

**Affiliations:** 1 Department of Cardiology, University Clinical Centre Maribor, Ljubljanska ulica 5, 2000 Maribor, Slovenia; husamfranjo.naji@ukc-mb.si; 2 Medical Faculty, University of Maribor, Taborska ulica 8, 2000 Maribor, Slovenia; dejan.dinevski@um.si

**Keywords:** sudden cardiac death, ECG, artificial intelligence, deep learning, machine learning, risk stratification

## Abstract

(1) Background and Objectives: Current risk stratification strategies for primary prevention of sudden cardiac death (SCD) have limited sensitivity and specificity. Artificial intelligence (AI) applied to electrocardiograms (ECGs) has emerged as a promising tool to predict the risk of future cardiac arrhythmias. This scoping review synthesizes evidence from original studies evaluating AI models trained on ECGs for risk stratification of SCD/malignant ventricular arrhythmias. (2) Materials and Methods: A comprehensive search of MEDLINE, Embase, Web of Science, Scopus and IEEE Xplore was conducted to identify peer-reviewed studies from inception to February 2026. Eligible studies included original investigations in which the model input was an ECG, recorded at baseline or during monitoring, and the outcome was either short-term or long-term SCD/malignant ventricular arrhythmia risk prediction. Extracted variables included study characteristics, ECG data, AI model data, model performance metrics, and the validation strategy. Risk of bias was assessed using PROBAST. (3) Results: Twenty studies met the inclusion criteria. High-risk cardiovascular subgroups (e.g., heart failure cohort, ICD cohort, etc.) or datasets from admitted patients, and conventional machine learning models or deep learning models were used in most studies. AI-ECG algorithms achieved moderate-to-high discriminative performance for identifying patients at an increased risk for imminent SCD/malignant ventricular arrhythmias (nine studies, AUROC ≈ 0.77–0.96) or future SCD/malignant ventricular arrhythmias (eleven studies, AUROC ≈ 0.66–0.94). However, multiple methodological limitations were identified, including limited sample sizes, susceptibility to overfitting, data imbalance-related bias, heterogeneity in dataset and endpoint definitions, inadequate external validation, and incomplete assessment and reporting of model calibration. (4) Conclusions: AI-ECG models demonstrate potential for risk stratification of SCD and malignant ventricular arrhythmias. However, the current evidence base is constrained by several methodological limitations, and further research is required to determine the clinical utility of AI-ECG for predicting SCD.

## 1. Introduction

Sudden cardiac death (SCD) remains an enormous medical and societal challenge, accounting for approximately 50% of all cardiovascular deaths and 11–20% of all deaths [1,2]. The incidence rates of SCD and out-of-hospital cardiac arrest (CA) were estimated at 250,000 cases (36.8–39.7 per 100,000 people) and 350,000 cases (47.8–57.9 per 100,000 people) yearly in the European Union [3]. Although the incidence rate of SCD decreased from 1990 to 2010, it has not shown any significant variations over recent years [3,4]. In up to 50% of cases, CA or SCD are the first manifestations of underlying cardiac disease [1,5]. Furthermore, the risk of SCD as a proportion of overall cardiovascular deaths may have increased recently [1,6]. Finally, the average survival following CA remains strikingly low, at approximately 5–10% for out-of-hospital CA, with little improvement in recent decades [7].

About half of the SCD cases are caused by ventricular tachycardia (VT) or ventricular fibrillation (VF) and could be theoretically prevented by a prophylactic implantation of a cardioverter–defibrillator (ICD) [2]. According to the current guidelines, primary prevention using an ICD is still mainly based on left ventricular ejection fraction (EF) and symptomatic evaluation [1,8,9]. However, the utility of EF in guiding ICD implantation for primary prevention in non-ischemic cardiomyopathy (NICM) has been questioned [10]. Moreover, a decline in the SCD risk in heart failure populations due to new pharmacotherapies and cardiac resynchronization therapy has been observed [11]. Finally, most of the SCD cases occur in populations with preserved ejection fraction [12]. Therefore, the majority of primary prevention patients do not receive an appropriate ICD therapy, while the majority of future SCD patients do not receive an ICD.

An electrocardiogram (ECG), an inexpensive, non-invasive, rapid and simple test, has a high potential as a screening modality for evaluating arrhythmic risk [1]. Abnormalities of ventricular depolarization (QRS fragmentation, etc.), repolarization (T wave inversion, etc.), substrate (LV hypertrophy, etc.) and autonomic system (heart rate variability, etc.) are all reflected on a 10 s 12-lead ECG [12,13]. However, subtle changes in electrophysiological properties of ventricular substrate may be overlooked during routine interpretation of ECGs [13]. Moreover, conventional ECG interpretation is limited by reliance on simple, linear, human-selected features, which may miss complex 3D waveform patterns and interactions across leads and time [12,14].

Artificial intelligence (AI), particularly deep learning (DL) methods, has transformed the analysis of high-dimensional physiological signals by enabling the identification of latent disease phenotypes invisible to the human eye [14]. AI-enabled ECG algorithms have demonstrated high diagnostic performance for detecting acute ischemia, left ventricular dysfunction and electrolyte disturbances, and have also been applied to predict new-onset atrial fibrillation from sinus rhythm ECGs [15,16]. Furthermore, AI-based algorithms have been recently used to predict SCD or CA from input sinus rhythm ECGs [17,18,19,20,21]. The earlier machine learning (ML) models relied on human-defined feature extraction (ECG measurements, heart rate variability data, etc.) [17,21], whereas the newer DL models automatically extract features from raw ECG waveforms [18,19,20]. Several algorithms have been trained and validated, and high model discrimination values (AUROC ~ 0.70–0.90) have been reported [17,18,19,20,21]. However, model performance relies on the heterogeneity of training datasets, ECG acquisition characteristics, endpoint definitions, and the prespecified prediction horizon for CA/SCD [14]. Moreover, interpretation of results and model comparison across reported studies are hindered by variability in external validation strategies, calibration assessment and adherence to reporting standards [22].

Therefore, a comprehensive synthesis of the available evidence is necessary to delineate the current state of the field, critically appraise methodological quality, summarize model discrimination and other performance metrics, identify key evidence gaps, and inform future AI-ECG-based risk stratification trials.

## 2. Materials and Methods

This scoping review was conducted in accordance with the PRISMA 2020 guidelines and adhered to standards for reporting AI-based diagnostic and prognostic studies (TRIPOD-AI) [23]. The protocol was prospectively developed before data extraction and predefined the research question, eligibility criteria, search strategy, data extraction protocol, and risk of bias assessment. The review protocol was registered prospectively on the Open Science Framework (registration DOI: 10.17605/OSF.IO/PYU3D) to enhance transparency.

### 2.1. Research Question

Among adults with available ECGs at baseline or during monitoring, how accurately do AI-based models predict future SCD or malignant ventricular arrhythmias (including sustained VT and VF (CA) and clinically relevant surrogate endpoints such as appropriate ICD therapies)?

### 2.2. Eligibility Criteria

#### 2.2.1. Inclusion Criteria

- Population: Adults (≥18 years) with ECG data recorded at baseline or during monitoring in any clinical setting (e.g., community cohorts, outpatient care, emergency department, intensive care unit, cardiomyopathy or inherited arrhythmia syndrome cohorts, ICD cohorts, etc.). Supraventricular arrhythmias on the index ECG and multiple ECGs per patient were allowed.
- Index test/intervention: Any supervised or unsupervised machine learning model (e.g., convolutional neural networks (CNNs), deep neural networks (DNNs), random forest models, support vector machines, etc.) trained on and applied to ECG data, including raw waveform signals and/or ECG-derived features (e.g., heart rate variability, repolarization/conduction markers, etc.).
- Comparator: Not mandatory. When present, comparators could include guideline-based risk stratification (LVEF-based ICD eligibility), clinical risk scores, clinician interpretation, or conventional ECG markers.
- Outcomes:
  - Prediction of short-term SCD/CA/malignant ventricular arrhythmia risk; risk stratification of patients for an event in the near future (time of the event < 14 days from the input ECG); identification of individuals at an increased risk for imminent CA/malignant ventricular arrhythmias.
  - Prediction of long-term SCD/CA/malignant ventricular arrhythmia risk; risk stratification of patients for an event in the distant future (time of the event > 14 days from the input ECG); identification of individuals at an increased risk for future CA/malignant ventricular arrhythmias.
  - Clinical implementation outcomes if AI-ECG was applied prospectively.
- Study design: Retrospective or prospective cohort studies, diagnostic accuracy and prognostic model studies, interventional or implementation trials.
- Publication: Full-length, peer-reviewed articles in English.
- Time frame: Inception to 13 February 2026.

#### 2.2.2. Exclusion Criteria

- Case reports, narrative reviews, editorials, letters, conference abstracts.
- Studies not evaluating SCD/malignant ventricular arrhythmia risk as an outcome.
- Studies not using electrocardiographic data as a model input (e.g., models based solely on clinical variables, imaging, or genomics).
- Studies using only intracardiac electrograms (from ICD or intracardiac mapping) as a model input.
- Animal studies or purely simulated datasets.

### 2.3. Search Strategy

The search was performed in MEDLINE (via PubMed), Embase, Web of Science Core Collection, Scopus and IEEE Xplore from inception to 13 February 2026. We combined controlled vocabulary and free-text terms for SCD, CA, VT, VF, ECG, AI, ML, DL, neural network, prediction and risk stratification, and adapted syntax to each database.

The core PubMed strategy was as follows:

(“Death, Sudden, Cardiac”[Mesh] OR “Heart Arrest”[Mesh] OR (sudden cardiac death[tiab] OR sudden cardiac arrest[tiab] OR SCD[tiab] OR SCA[tiab] OR “ventricular fibrillation”[tiab] OR “ventricular tachycardia”[tiab] OR “electrical storm”[tiab])) AND (“Electrocardiography”[Mesh] OR electrocardiogram*[tiab] OR ECG[tiab] OR EKG[tiab] OR “12-lead”[tiab] OR Holter[tiab] OR “ambulatory ECG”[tiab]) AND (“Artificial Intelligence”[Mesh] OR “Machine Learning”[Mesh] OR “Deep Learning”[Mesh] OR artificial intelligence[tiab] OR machine learning[tiab] OR deep learning[tiab] OR neural network*[tiab] OR CNN[tiab]) AND (predict*[tiab] OR prognos*[tiab] OR risk strat*[tiab] OR model*[tiab] OR score*[tiab]).

We added filters for humans, adults, English language, and excluded obvious non-research article types. The search was supplemented by backward citation tracking of key primary studies and existing systematic reviews.

### 2.4. Study Selection

Two reviewers independently screened all titles and abstracts for relevance, which was followed by a full-text assessment against the eligibility criteria. Disagreements were resolved by a consensus.

### 2.5. Data Extraction

From each included study, two reviewers independently extracted data using a predefined extraction form. Extracted variables included:

- Study characteristics: year, country, setting, design, sample size, inclusion/exclusion criteria, follow-up.
- ECG data: lead configuration, duration, sampling frequency, filters.
- AI model: architecture (CNN, DNN, support vector machines, random forest models, etc.), input type (raw waveform signals, heart rate variability, conduction/repolarization markers), training/validation cohort splits, availability of explainability methods.
- Validation strategy: internal and/or external validation.
- Reference standard for outcome ascertainment: SCD adjudication methods, cardiac arrest documentation, ventricular arrhythmia and appropriate ICD therapy definitions.
- Prediction horizon (including windowing strategies where applicable).
- Model performance metrics: AUC/AUROC, AUPRC, sensitivity, specificity, PPV/NPV, calibration metrics (if reported).
- Clinical outcomes or implementation outcomes if reported.
- Funding sources and potential conflicts of interest.

### 2.6. Risk of Bias and Applicability

Because the primary outcome is risk prediction/diagnostic performance, the Prediction model Risk of Bias Assessment Tool (PROBAST—version of 15 May 2019) was used to assess the risk of bias and applicability for each included study. Domains included participant selection, predictors, outcome definition, and analysis. Discrepancies were resolved by a consensus.

### 2.7. Data Synthesis and Analysis

Given the marked heterogeneity in datasets/populations, AI architectures, study outcomes, and prediction horizons, a quantitative meta-analysis was not planned. A structured narrative synthesis was prespecified. The heterogeneity of included studies was assessed qualitatively by visual inspection of forest plots. Two major groups of outcomes were identified (short-term vs. long-term SCD/malignant ventricular arrhythmia risk prediction).

## 3. Results

### 3.1. Study Selection

**Figure 1:**
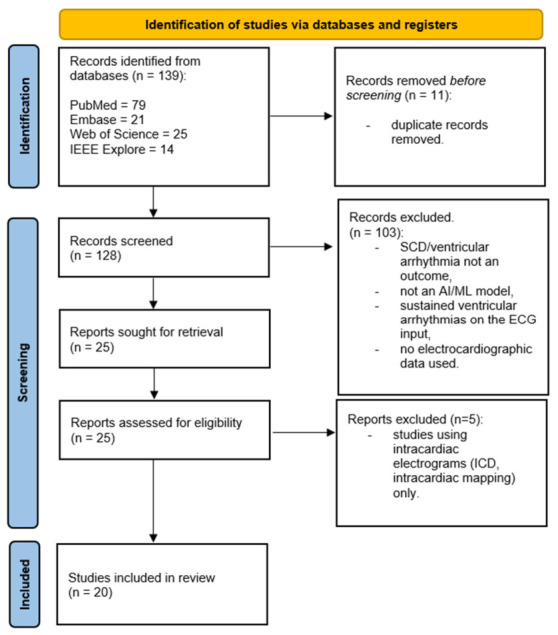
PRISMA flow diagram: PRISMA—preferred reporting items for systematic reviews and meta-analyses, SCD—sudden cardiac death, AI—artificial intelligence, ML—machine learning, ECG—electrocardiogram, ICD—implantable cardioverter–defibrillator.

The database search and citation tracking identified 139 potentially relevant articles. After removal of duplicates and two-stage screening (title/abstract and full text), 20 studies met all predefined eligibility criteria. These studies evaluated AI models applied to ECGs to predict short-term or long-term risk of SCD or malignant ventricular arrhythmias across a wide range of healthcare settings. The screening process is summarized by the PRISMA flow diagram (Figure 1).

### 3.2. Study Characteristics

#### 3.2.1. Settings and Patient Populations of the Included Studies

Across the 20 studies, patient populations varied widely. Training and validation cohorts included datasets from admitted patients [18], encompassing emergency department triage cohorts [24], and intensive care unit cohorts [17,21]. Ambulatory patient datasets were employed in some of the studies [25,26]. Several reports utilized publicly available online datasets [26,27,28,29]. High-risk cardiovascular subgroups, such as dilated cardiomyopathy cohorts [30,31], phospholamban cardiomyopathy patients [32], heart failure patients [33], and ICD cohorts [19,34,35], were often included. Sample sizes ranged from less than a hundred ECGs [21,27] to tens or hundreds of thousands of included ECGs [18,26]. Mean patient age typically ranged from 60 to 75 years, and the vast majority of studies used patients at an increased baseline risk for SCD or malignant ventricular arrhythmias [17,18,19,20,21,25,26,27,28,29,30,31].

#### 3.2.2. ECG Modalities

Several studies employed continuous single-lead ECG monitoring, acquired via ambulatory recording platforms or in-hospital monitoring systems [17,21,24,25,27,36]. Nevertheless, the majority of studies used 10 s, 12-lead resting sinus rhythm ECGs in both training and testing cohorts [18,19,20,31,32,33,34,35,37,38]. Serial 12-lead ECG recordings were seldom reported [19,21,27]. All studies documented sinus rhythm or supraventricular arrhythmias on the index ECGs; no study included patients with sustained ventricular arrhythmias in model input ECG recordings.

#### 3.2.3. AI Model Types

The included studies reported both conventional ML models that rely on manually engineered feature extraction, and DL approaches that perform automated feature learning directly from raw ECG data.

Conventional ML models included:

- Support vector machines (SVMs) [24,27,30].
- K-nearest neighbors algorithms (KNNs) [17,27].
- Decision tree-based algorithms (DTs) [21,27].
- Random forest models (RFs) [17,27,38].
- An extreme gradient boosting model (XGB) [38].

DL models included:

- Convolutional neural networks (CNNs) [18,20,25,26,29,33,37].
- Deep neural networks (DNNs) [19,32,34].
- Recurrent neural networks (RNNs), in combination with CNNs [28,33].
- K-means and hierarchical clustering [35].

#### 3.2.4. Outcomes and Validation

Two major categories of study outcomes were identified:

- Short-term SCD/CA/malignant ventricular arrhythmia risk prediction (time of the event < 14 days from the index ECG; Table 1) [17,18,21,24,26,27,28,29,36].
- Long-term SCD/CA/malignant ventricular arrhythmia risk prediction (time of the event > 14 days from the baseline ECG, Table 2) [19,20,25,30,31,32,33,34,35,37,38].

Internal validation was reported in 14 out of 20 studies and external validation was documented in five out of 20 studies (Table 1 and Table 2). Large multi-center databases were used as testing cohorts in several studies [18,19,20,21,33,38].

### 3.3. Performance of AI Models

**Figure 2:**
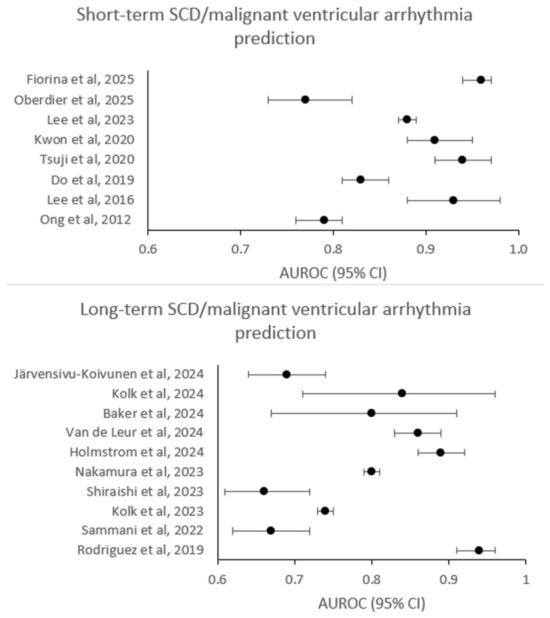
Forest plots of outcome measures (AUROC and 95% CI). Forest plots of outcome measures (AUROC and 95% CI): AUROC—area under a receiver operating characteristic curve, CI—confidence interval, SCD—sudden cardiac death [17,18,19,20,21,24,25,26,28,29,30,31,32,33,34,36,37,38].

The discriminative performance of AI-based models is summarized in Figure 2.

#### 3.3.1. Short-Term SCD/CA/Malignant Ventricular Arrhythmia Risk Prediction

Nine studies evaluated AI models to predict short-term SCD/CA/malignant ventricular arrhythmia risk based on a sinus rhythm ECG input, and to identify individuals at an increased risk for imminent ventricular arrhythmias (Table 1).

- Ong et al., 2012 [24]: AUROC 0.78 was achieved by an SVM for predicting CA within 72 h in hospitalized patients (*n* = 925). The algorithm evaluated heart rate variability (HRV) on continuous single-lead ECG recordings.
- Lee et al., 2016 [36]: Artificial neural network based on HRV analysis achieved AUROC 0.93 for 1 h VT prediction in an ICU cohort (*n* = 82).
- Lai et al., 2019 [27]: Several conventional ML models including KNN, DT, SVM and RF were trained and tested on a very small publicly available dataset (*n* = 46). A 99.59% accuracy was reported using an RF algorithm to predict SCD within 30 min.
- Do et al., 2020 [17]: RF model accurately predicted CA within 3 h in ICU patients (*n* = 1874) with AUROC 0.83. Prespecified ECG measurements (QRS amplitude and duration, corrected QT interval, etc.) were analyzed.
- Tsuji et al., 2020 [28]: AUROC 0.94 was reported using an RNN trained and tested on a very small dataset (*n* = 20). The algorithm was based on HRV analysis and used to predict VF within the next minute.
- Kwon et al., 2020 [18]: A CNN was trained and tested on ≈ 47,000 10 s 12-lead ECGs in a large multi-center trial. The algorithm achieved AUROC 0.91 for predicting CA within 24 h. Prespecified external validation was performed.
- Lee et al., 2023 [21]: A DT-based ML model accurately predicted CA within 24 h in ICU patients (*n* = 4821) with AUROC 0.88 and AUPRC 0.11. The algorithm analyzed HRV features.
- Oberdier et al., 2025 [29]: A CNN was trained and tested on a publicly available dataset (*n* = 1326) and was used to predict CA within 24 h. The model achieved AUROC 0.77.
- Fiorina et al., 2025 [26]: A large publicly available international ambulatory dataset (*n* ≈ 250,000) was used to train and test a CNN to predict sustained VT within 13 days. The model achieved AUROC 0.96 and AUPRC 0.30 and was externally validated.

Across different settings and patient cohorts, AI models predicted short-term SCD/CA/malignant ventricular arrhythmia risk with AUROC ≈ 0.77–0.96. However, several studies included a limited number of ECG recordings. Furthermore, external validation was rarely performed.

#### 3.3.2. Long-Term SCD/CA/Malignant Ventricular Arrhythmia Risk Prediction

Eleven studies evaluated AI models to predict long-term SCD/CA/malignant ventricular arrhythmia risk based on a sinus rhythm ECG input, and to identify individuals at an increased risk for future ventricular arrhythmias (Table 2).

- Rodriguez et al., 2019 [30]: AUROC 0.94 was achieved by an SVM for predicting SCD/CA in a DCM cohort (*n* = 140) over a mean follow-up period of 28 months. The AI-based model evaluated HRV on continuous single-lead ECG recordings.
- Sammani et al., 2022 [31]: In a cohort of 695 DCM patients, an explainable DNN was used to predict SCD/sustained VT/appropriate ICD therapy within a median follow-up of 51 months. The model achieved AUROC 0.67.
- Kolk et al., 2023 [19]: A dynamic ML model using longitudinal ECG data from ≈ 3000 ICD patients was tested to predict sustained ventricular arrhythmias within 3 months. The dynamic ML model achieved AUROC 0.74, outperforming a static ML model.
- Shiraishi et al., 2023 [33]: In a prospective multi-center study, a DL model (CNN + RNN) was developed to predict SCD/appropriate ICD therapy within 36 months amongst patients hospitalized for heart failure decompensation (*n* = 2559). The multimodal model based on ECG-AI and clinical characteristics achieved AUROC 0.66 and was externally validated.
- Nakamura et al., 2023 [37]: A CNN was trained and tested on a Brugada syndrome cohort (*n* = 157) to predict VF within a median follow-up of 42 months. The algorithm achieved AUROC 0.80.
- Holmstrom et al., 2024 [20]: In a community cohort of 3835 patients, a CNN was used to predict SCD within a median follow-up of 20 months. The model achieved AUROC 0.89 and was externally validated.
- Van de Leur et al., 2024 [32]: An explainable DNN was developed to predict SCD/sustained VT/appropriate ICD intervention within 60 months in a cohort of 679 patients with phospholamban cardiomyopathy. The algorithm achieved AUROC 0.86 and AUPRC 0.27.
- Barker et al., 2024 [25]: AUROC 0.80 was achieved by a CNN in an ambulatory dataset (*n* = 270; 10 s 3-lead ECG input). The model was used to predict CA/sustained VT over a median follow-up of 19 months.
- Kolk et al., 2024 [34]: In a cohort of 289 NICM patients with ICD, a multimodal ML algorithm (ECG features, LGE MRI scans, clinical characteristics) was used to predict CA/sustained VT/appropriate ICD intervention within 12 months. The multimodal model achieved AUROC 0.84 and AUPRC 0.31, outperforming an ECG-AI-only algorithm, and was externally validated.
- Järvensivu-Koivunen et al., 2024 [38]: Conventional ML algorithms (XGB and RF) were developed to predict SCD or appropriate ICD therapy within a median follow-up of 5 years in a post-ACS cohort (*n* = 8568). The models evaluated ECG measurements (QRS axis and amplitude, etc.) and achieved AUROC ≈ 0.65–0.70.
- Tateishi et al., 2025 [35]: Unsupervised ML algorithms (K-means and hierarchical clustering) were used to predict appropriate ICD therapy within a median follow-up of 98 months in an ICD cohort (*n* = 200). Hierarchical clustering achieved a silhouette coefficient 0.58.

Across eleven studies performed in different settings, AI-based models achieved AUROC ≈ 0.66–0.94 for long-term SCD/CA/malignant ventricular arrhythmia risk prediction. Several studies included a limited number of patients; high-risk cardiovascular subgroups were often reported. External validation was rarely performed.

### 3.4. Risk of Bias and Applicability

The PROBAST tool was used to assess the risk of bias and applicability for each included study (Table S1, Supplementary Materials).

- Participant selection: Several AI-based models were trained on insufficient sample sizes. Furthermore, the majority of reports focused on patients who were at a high risk for SCD/malignant ventricular arrhythmias. Additionally, retrospective single-center patient cohorts were used in most studies; prospective international cohorts were rarely reported. Finally, datasets from high-income countries were often used; data from low-resource countries is limited.
- Predictors: Datasets were not always split into training, validation and testing cohorts. Using multiple ECGs per patient was variably reported.
- Outcomes: Outcomes (SCD/CA/malignant ventricular arrhythmia) were verified in all studies. However, appropriate ICD therapy was often used as a surrogate for SCD/malignant ventricular arrhythmia.
- Analysis: Although internal validation was performed in most studies, external validation was reported in only 5/20 studies. Model calibration and decision-curve reporting were inconsistent. The number of participants with the outcome was limited in several studies due to a small testing cohort and a low incidence of SCD/malignant ventricular arrhythmias. Therefore, these studies were susceptible to overfitting and class imbalance-related bias.

Overall, risk of bias was high in thirteen studies, low in four studies, and unclear in three studies. Concerns regarding applicability were present in 5/20 reports. Although the majority of studies were applicable to the review question, major risk of bias was possible in 75% of the included reports.

## 4. Discussion

This scoping review synthesized evidence from studies that applied AI-based models to ECGs recorded at baseline or during monitoring to predict SCD or malignant ventricular arrhythmias. Across 20 studies with different settings and patient populations included in this review, the principal finding is consistent. AI-ECG models demonstrate potential for identifying patients at an increased risk for imminent SCD/malignant ventricular arrhythmias (nine studies, AUROC ≈ 0.77–0.96) or future SCD/malignant ventricular arrhythmias (eleven studies, AUROC ≈ 0.66–0.94) [17,18,19,20,21,24,25,26,27,28,29,30,31,32,33,34,35,36,37,38]. However, several studies reported low-quality models built on ad hoc datasets and trained on insufficient sample sizes [27,28,30,35,36,37]. Furthermore, external validation and model calibration were inconsistently reported [18,19,20,26,33]. Additionally, the explainability of DL models was not always assessed [18,29,31,32,37]. Therefore, large, prospective, multi-center studies are needed to establish generalizability and determine the clinical utility of AI-based ECG algorithms for risk stratification of SCD and malignant ventricular arrhythmias.

### 4.1. Electrical Instability and ECG Substrate in SCD

People who suffer a CA or SCD form a heterogeneous population with diverse arrhythmogenic substrate characteristics (coronary artery disease, structural heart disease, primary electrical disease). Although many individuals have anatomic (fibrosis, hypertrophy, etc.) and functional (ion channel dysregulation, gap junction remodeling) substrates conducive to developing life-threating ventricular arrhythmias and many patients have transient events that could predispose them to the initiation of VT/VF, only a minority of patients develop SCD [12]. According to Coumel’s triangle of arrhythmogenesis, the electrophysiological properties of both the arrhythmia substrate and trigger factors vary in time due to the influence of modulation factors (the autonomic nervous system, electrolyte disbalance, inflammation, etc.) [39]. Furthermore, individuals with a genetic predisposition often require a second-hit to trigger arrhythmias [40]. These features create a dynamic target for risk stratification and complicate both clinical decision-making and model development. Even though the relative risk is highest among patients with a history of cardiac disease or a genetic predisposition, most of the SCD cases occur in the general population with a preserved ejection fraction (EF) [12]. Nevertheless, the CAD is at least indirectly responsible for up-to 75–80% of SCD cases, especially in patients over the age of 50 years [1,41]. Finally, cardiomyopathies and primary electrical diseases, associated with abnormalities in impulse conduction, cardiomyocyte action potential duration and cardiac repolarization dispersion, represent an important cause of SCD, particularly in the young [1].

The association between left ventricular hypertrophy and sudden death was first reported in the Framingham cohort in the 1970s [42] and acquired QT interval prolongation was identified as a risk marker for sudden death in 1978 [43]. Thenceforth, a plethora of studies has examined the value of different ECG-derived risk parameters in predicting SCD, either in the general population or in patients with a history of cardiac disease [13]. Resting heart rate and heart rate variability, abnormalities of depolarization (pathologic Q waves, QRS duration, QRS fragmentation, increased and reduced myocardial voltage, intra-atrial conduction delay, etc.), abnormalities of repolarization (QTc/JTc, QT dispersion, T-peak to T-end interval, QRS-T angle, T wave alternans, etc.), and cardiac arrhythmias (atrial fibrillation, non-sustained VT, etc.) have all been reported to have prognostic value for SCD prediction in different clinical settings [12,13]. However, their practical value is limited. First, conventional ECG-derived SCD risk stratification is based on studies using high-risk patient cohorts, and reports with an insufficient sample size and a limited follow-up duration [12]. Second, as the risk of SCD in the general population is low, the absolute predictive value of a single parameter test will also be low [1,44]. Prospective studies determining which parameter combinations provide strong risk predictors are rare. In one report, an ECG-based risk score predicted SCD with AUROC 0.74 in a community-based cohort within 2 years [45]. Third, conventional ECG interpretation is focused on simple, linear, human-selected features, which may miss complex 3D waveform patterns and interactions across leads and time [12,14].

### 4.2. ECG-AI Model Types and Model Explainability

AI-based algorithms have improved diagnostic accuracy and efficiency in detecting electrocardiographic abnormalities through automated feature extraction in large datasets [14]. According to feature extraction, AI-based models can be broadly classified into conventional ML models that rely on manually engineered feature extraction and DL algorithms that learn hierarchical representations directly from the input signal [46]. Feature-based ML models are typically hypothesis-driven and depend on human-defined rules, including standard ECG measurements and indices derived from continuous ECG monitoring [46]. In this scoping review, 10/20 studies employed different conventional ML algorithms (RF, DT, KNN, SVM, XGB) to predict SCD-related outcomes [17,19,21,24,26,27,30,34,36,38]. In the majority of studies, epochs were constructed from continuous ECG monitoring data and ML models based on heart rate variability indices were used to predict the short-term risk of SCD/malignant ventricular arrhythmia [21,24,26,30,36]. Three reports utilized several ML algorithms to predict the outcomes directly from standard ECG measurements (QRS amplitude, QTc interval, ST segment, cardiac arrhythmias, etc.) [17,27,38]. Furthermore, a conventional multimodal ML model, using MRI data, ECG features and clinical characteristics, and a dynamic ML model, using longitudinal ECG data, were constructed to predict the long-term SCD/malignant ventricular arrhythmia risk within an ICD cohort [19,34]. Both studies also employed a DL model for ECG feature extraction.

Conversely, DL algorithms are empirical and are able to predict outcomes directly from raw ECG waveforms [14]. DL models were developed in 12/20 studies included in this scoping review and were most often used for a long-term SCD/malignant ventricular arrhythmia risk stratification [18,19,20,25,28,29,31,32,33,34,35,37]. DL-based algorithms have traditionally been regarded as “black-box” models, providing limited interpretability regarding which ECG waveform features drive their predictions. In the absence of explainability, clinicians may struggle to trust AI-derived risk predictions, thereby constraining widespread adoption in clinical practice. Consequently, several studies employed interpretability techniques to localize the area on the ECG that contributed most strongly to model outputs and to improve model transparency [18,29,31,32,37]. Saliency mapping revealed that a CNN focused mostly on the QRS complex and the T wave area, which correspond to ventricular depolarization and repolarization, predicts a CA within 24 h [18]. In the aforementioned report, cardiac arrest ECGs had prolonged QRS durations and QTc intervals, more rightward T wave axes and greater resting heart rates. Moreover, gradient-weighted class activation mapping analysis (Grad-CAM) revealed a general emphasis on the QRS complex in another study using a CNN to predict a CA within 24 h [29]. Finally, explainable DNNs called variational autoencoders were used to compress ECG data into a set of different generative ECG factors [31,32]. In one report, five generative ECG factors (PR interval, P wave amplitude, right bundle branch delay, R wave axis and QRS-T amplitude) were associated with the prediction of SCD/malignant ventricular arrhythmia in a DCM cohort [31]. These findings support the hypothesis that DL-based networks might exploit physiological markers of impaired ventricular conduction and repolarization. However, large prospective clinical trials using novel explainable AI approaches, such as attention mechanisms, occlusion-based techniques and Grad-CAM, are warranted to truly explain the model outcome prediction [47].

### 4.3. Model Discrimination and Model Architecture-Outcome Alignment

Across the included studies, model discrimination was generally assessed using standard classification metrics, such as AUC. AUROC ≈ 0.77–0.96 was reported across nine studies for short-term SCD/malignant ventricular arrhythmia risk stratification. AUROC 0.83 was achieved using an RF model to predict a CA within 3 h in a cohort of 1874 ICU patients [17]. The ML model was based on prespecified ECG measurements (QRS duration, RR interval, ST segment changes, atrial fibrillation and AV block). In another study, a CNN was trained and tested on ≈ 47,000 10 s 12-lead ECGs [18]. The DL model demonstrated AUROC 0.91 for 24 h CA prediction. Furthermore, patients who were identified as high-risk by the algorithm were significantly more likely to develop a CA within 2 weeks compared with those classified as low-risk (5.74% vs. 0.33%, *p* < 0.001). In a recent study employing a large ambulatory dataset (*n* ≈ 250,000), a CNN was trained on 24 h single-lead recordings to predict sustained VT within 13 days [26]. The algorithm achieved AUROC 0.96 and outperformed a multivariable logistic regression model based on standard continuous monitoring ECG measurements. The model discrimination improved at shorter prediction horizons (2–3 days). Moreover, AI-based models achieved AUROC ≈ 0.66–0.94 for long-term SCD/CA/malignant ventricular arrhythmia risk prediction. In one report, several conventional ML algorithms were developed to predict SCD in a post-ACS cohort with ≈ 8500 patients within five years of follow-up [38]. AUROC 0.693 was achieved using the XGB model. The most important ECG features identified by the AI model were QRS duration, intraventricular conduction disease, QTc interval and the presence of ventricular extrasystoles. In community SCD cohorts, a CNN was trained and tested on 3835 patients [20]. The DL model predicted SCD with AUROC 0.89 within 20 months and performed significantly better than the conventional electrical risk score (AUROC 0.712) [45]. Finally, the addition of the clinical variables into the ECG-AI index improved the model discrimination. A dynamic ML model (RF-SLAM) using baseline clinical parameters and baseline and dynamic ECG variables was developed to predict sustained VT within 3 months in a cohort of ≈ 3000 ICD patients [19]. The ECG data was obtained using a variational autoencoder. The algorithm achieved AUROC 0.74, outperforming a static ML model (AUROC 0.64).

A central finding of this scoping review is that model architecture should be interpreted in relation to the intended prediction horizon and clinical use case. The included studies do not represent a single homogeneous class of AI-ECG models; rather, they can be separated into at least two conceptually distinct groups. First, short-term prediction models, usually targeting SCD/malignant ventricular arrhythmias within minutes to days, often used continuous or ambulatory ECG signals and frequently relied on HRV-derived features or dynamic waveform information [17,18,21,24,26,27,28,29,36]. These models appear suited to detecting transient changes in autonomic tone, conduction instability, repolarization dynamics, or acute physiological deterioration [17,21]. Their likely role is therefore not broad population screening, but near-term warning in monitored environments such as emergency departments, ICUs, or inpatient telemetry systems. Second, long-term prediction models, targeting events over months to years, more often used standard 10 s 12-lead ECGs, deep learning architectures, or multimodal models combining ECG features with clinical or imaging data [19,20,25,30,31,32,33,34,35,37,38]. These models are better conceptualized as tools for detecting stable or slowly evolving arrhythmogenic substrate characteristics rather than imminent triggers [19,20]. Therefore, their potential clinical role is the refinement of SCD risk stratification in selected populations, such as patients with heart failure, cardiomyopathy or primary electrical disease; post-acute coronary syndrome cohorts; or ICD populations. Importantly, these two model categories should not be compared against each other, because they address different biological targets, prediction windows, and event prevalences.

### 4.4. Limitations of the Current Data

Despite the promising results achieved by AI-based models, it is crucial to consider the limitations of the included studies. First, only 9/20 studies reported using datasets with >1000 patients [17,18,19,20,21,26,29,33,38] and only 2/20 reports included more than 10,000 patients [18,26]. Inadequate datasets are often not representative of the target population and setting [22,48]. Therefore, models trained on an insufficient sample size tend to perform worse in external cohorts. Small training samples produce large model instability, which yields considerable uncertainty in individual predictions, and increases the likelihood of generating poorly calibrated predictions [48].

Second, the proportion of patients who developed SCD/malignant ventricular arrhythmia was low in the majority of studies. Data imbalance is an inherent problem in the study of rare outcomes, such as SCD, and occurs when the number of observations per class is not equally distributed [49]. Imbalanced datasets may negatively impact model robustness and can lead to biased model predictions and poor performance on minority classes [22,23]. Although balanced datasets were reported in a few studies, the overall sample size in these studies was small, as they included less than 1000 patients [25,27,30,36]. The only study which adequately dealt with data imbalance and retained a relatively large sample was Holmstrom et al., which used a cohort of 3835 patients, 2510 of whom suffered SCD [20]. Moreover, the standard classification metrics (AUROC), which were used as a primary metric of model discrimination in most of the included studies, do not adequately represent model performance when datasets are imbalanced [50]. Theoretically, an accuracy of 99% would be achieved using a model with an all-negative prediction in an imbalanced dataset with a 1% prevalence of SCD. Therefore, alternate performance metrics, such as F1 score and precision-recall curves (AUPRC), should be reported in addition to c-statistic [50]. In the minority of the included studies which reported alternate performance metrics, AI-based models achieved AUPRC ≈ 0.10–0.30 and F1 score ≈ 80% [20,21,25,26,32,34,37].

Third, heterogeneity in datasets and endpoint definitions both remain a major limitation. Subpopulations with a high baseline risk for SCD were included in most of the reports. While several studies used patients with a specific structural cardiac disease (dilatative cardiomyopathy, phospholamban cardiomyopathy) [30,31,32] or primary electrical disease (Brugada syndrome) [37], other reports focused on diverse cohorts with an increased risk for SCD (heart failure patients, ICD cohorts, ICU patients, etc.) [19,21,24,33,38]. Accordingly, these algorithms should only be used in the target groups, as they would overpredict SCD in the general population. Finally, heterogeneity across datasets precludes head-to-head comparisons of model discrimination metrics and renders a quantitative meta-analysis infeasible [23]. Nevertheless, the included studies variably used SCD, CA, sustained VT, and appropriate ICD therapy as primary endpoints. Although these outcomes are related, they are not equivalent. Ventricular tachyarrhythmias that prompt appropriate ICD intervention may be non-sustained, self-terminating, or hemodynamically tolerated, and therefore would not necessarily have resulted in SCD/CA in the absence of device therapy [1]. Consequently, the use of appropriate ICD therapy as an endpoint may overestimate the true SCD event rate and inflate event counts relative to adjudicated fatal or near-fatal arrhythmic outcomes [19,31,32,33,34,35].

Fourth, although 14/20 studies performed internal validation, external validation was reported in only 5/20 of the included studies (Table 1 and Table 2). Splitting of the dataset into training and internal validation cohorts is crucial to prevent overfitting [22]. Overfitting occurs when a model learns the idiosyncratic noise in the training data rather than the discriminative features, resulting in an inflated performance during training but degraded performance when evaluated on previously unseen data [49]. Accordingly, metrics of the internal validation cohort best represent true model discrimination and performance. Furthermore, ML models developed and evaluated on non-representative datasets may struggle to perform reliably when applied to external populations, reflecting differences in ECG acquisition data, demographic composition, comorbidity profiles, and other setting-specific characteristics [51]. In contrast, several studies that leveraged large, multi-institutional datasets for model development reported more robust generalizability, with AUROC values remaining above 0.80 across independent test cohorts [18,20,26].

Fifth, model calibration was reported in only 3/20 of the included studies [19,33,34]. The process of adjusting model parameters to better align predicted probabilities with observed event rates is a critical component of developing reliable and robust AI-based prediction models [52]. Models trained in high-risk groups may produce systematically inflated absolute risk estimates when applied to lower-risk populations, such as unselected community cohorts [23]. Consequently, AI-based algorithms may be most appropriately applied in populations with SCD prevalence similar to the development cohort unless explicit model recalibration is performed [17]. In two reports, model calibration was assessed using calibration plots, which compared the predicted probabilities against the observed event rates in grouped data [19,34]. Calibration curve visualization showed overestimated SCD prediction in one study and a good agreement between the predicted risks and the observed outcomes in the second study. Finally, no significant difference between observed and predicted values (*p* = 0.11), implicating a good fit, was noted using the Hosmer–Lemeshow goodness-of-fit test in one report [33].

Several methodological gaps are especially relevant in the context of AI-ECG prediction of SCD and malignant ventricular arrhythmias. These include the low incidence and time-varying nature of SCD risk, substantial heterogeneity in datasets and endpoint definitions, incomplete performance reporting, a lack of calibration, and insufficient external validation. All of these limitations are likely to affect the real-world discrimination, performance and overall clinical utility of the proposed algorithms. Therefore, the current body of evidence should be interpreted primarily as hypothesis-generating, and adequately powered prospective trials will be required to establish the true clinical value of AI-ECG for SCD prediction.

### 4.5. Implications for the Future

As emphasized in the 2025 EHRA and HRS scientific statement, regarding the state of the art of AI in clinical electrophysiology, AI-enabled ECG models constitute a promising approach for arrhythmia prevention and risk stratification. However, robust validation is required before they can be recommended for routine clinical use [53]. On the basis of the current evidence, future AI-ECG studies for SCD prediction should begin by defining the intended clinical use cases. Models designed for short-term warning should be developed and evaluated separately from models designed for long-term risk stratification, as these applications differ with respect to ECG inputs, prediction horizons, and the clinically acceptable false-positive rate. Short-term models should be evaluated in care environments with continuous patient ECG monitoring and should report time-dependent performance at clinically actionable intervals [17]. Long-term models should be benchmarked against guideline-based risk markers and should demonstrate incremental prognostic value beyond contemporary risk stratification approaches (LVEF, clinical variables, etc.) [1]. Future research should prioritize adequately powered, prospective, multi-center, randomized studies that leverage large, heterogeneous datasets to develop and evaluate AI-ECG models for either short-term or long-term SCD/malignant ventricular arrhythmia risk stratification. Furthermore, large heterogeneous training datasets could improve model discrimination and generalizability across various demographic groups. Moreover, a 12-lead ECG provides a snapshot of the cardiac electrical activity at the moment of the acquisition and may not adequately capture the dynamic nature of an individual’s arrhythmic risk [39]. Therefore, integrating ECG-based AI outputs with complementary modalities, such as cardiac imaging, circulating biomarkers, and genetic data, may enable multimodal risk models with improved predictive performance. A multimodal ML model using MRI data, ECG waveform and clinical characteristics outperformed a purely ECG-AI model in one study [34]. AI ECG models have been increasingly designed for high sensitivity to act as screening tools, often at the expense of reduced specificity and positive predictive value (PPV), particularly in low-prevalence populations, such as SCD. Deployment of such models in unselected populations could therefore generate a substantial burden of false-positive alerts, increasing downstream testing and potentially overwhelming clinical workflows. Accordingly, future studies should carefully define intended-use populations and operating thresholds, and explicitly evaluate trade-offs between sensitivity and specificity in relation to clinically actionable pathways and resource constraints [14,51].

DL models are able to preserve the complexity in multidimensional data, handle a large number of variables, and predict outcomes directly from ECG waveforms [14]. Consequently, DL algorithms were used in more than half of the included reports and will most likely be employed in the majority of future studies. However, the interpretability and explainability of DL models remain a major concern [14,54]. Although explainable DNNs and sensitivity analyses have been employed in some studies to improve the explainability of the developed models, not every feature in the latent space has a direct one-to-one correspondence with a known ECG finding [54]. Therefore, additional research into the explainability of AI-based models is warranted to facilitate implementation into the clinical practice. Furthermore, novel explainable AI techniques should be employed in future studies. Algorithms, such as attention mechanisms and Grad-CAM, are specifically designed to enhance interpretability in ECG-based DL and highlight exactly which part of the ECG is important for establishing the diagnosis [47]. Nevertheless, the field of ECG-AI has been characterized by low-quality models, trained on insufficient sample sizes [22]. Although recent studies have increasingly adopted the rigorous methodological standards for AI model development, susceptibility to overfitting and bias related to class imbalance remain an issue in the included reports. Moreover, model calibration and external validation were inconsistently reported. Consequently, future research should adhere to contemporary standards for reporting AI-based diagnostic and prognostic studies [23]. We recommend prespecified protocols, adequate sample-size justification, clear handling of multiple ECGs per patient, robust missing-data strategies, and prespecified internal and external validation. Performance reporting should include AUROC, AUPRC, calibration, clinically interpretable risk thresholds, and decision-curve analysis [22,23,53]. Furthermore, to reduce class imbalance-related bias, future studies may utilize various data-based methods, such as undersampling (e.g., cluster-based undersampling), oversampling (e.g., synthetic minority oversampling—SMOTE), and data transformation techniques (e.g., loss function-based methods using class weighting). Finally, existing AI-based models should be tested against each other and across diverse populations to prove the reproducibility of reported results.

## 5. Conclusions

This scoping review provides an overview of the current state-of-the-art regarding ECG-AI prediction models for SCD. AI applied to ECGs, acquired at baseline or during monitoring, demonstrates potential as a non-invasive tool for either short-term or long-term SCD/malignant ventricular arrhythmia risk stratification. However, the current body of evidence is constrained by several methodological limitations and should therefore be interpreted primarily as exploratory and hypothesis-generating rather than confirmatory. Future trials will determine whether AI-ECG models have a role in primary prevention of SCD and whether they can become a part of everyday clinical care.

## Figures and Tables

**Table 1:** Short-term risk stratification studies.

Study (Year of Publication)	Study Type	Population and Setting	Number of Patients/Recordings	ECG Modality and Analyzed Data	Outcome	AI Model Type and Included Parameters	Validation	Model Discrimination	Other Performance Metrics
Ong et al., (2012) [24]	Single-center, prospective observational study	Hospitalized patients, emergency department	925 patients (43 patients with cardiac arrest)	30 min single-lead ECG recordings, HRV	Cardiac arrest prediction within 72 h	SVM (HRV + clinical variables)	Internal validation	AUROC 0.78	Sensitivity 81.4%, specificity 72.3%, PPV 12.5%, NPV 98.8%
Lee et al., (2016) [36]	Single-center, retrospective observational study	Hospitalized patients, coronary care unit	82 patients (41 patients with VT)	5 min single-lead ECG recordings, HRV	VT prediction within 1 h	Artificial neural network (HRV + respiratory rate variability)	Not reported	AUROC 0.93	Sensitivity 88.2%, specificity 83.3%, PPV 83.3%, NPV 87.5%
Lai et al., (2019) [27]	Single-center, retrospective observational study	A publicly available dataset	46 recordings (30 recordings with cardiac arrest)	Serial 1 min 12-lead ECG recordings, advanced repolarization intervals and conduction-repolarization markers	SCD prediction within 30 min	Conventional ML models (KNN, DT, SVM and RF), ECG measurements	Not reported	/	Sensitivity 99.75%, specificity 99.04%, accuracy 99.59% (RF model)
Do et al., (2019) [17]	Single-center, retrospective observational study	Hospitalized patients, intensive care unit	1874 patients (91 patients with cardiac arrest)	3 h multiple-lead ECG recordings, QRS amplitude and duration, QTc interval, RR interval, ST segment, arrhythmias	Cardiac arrest prediction within 3 h	Conventional ML models (logistic regression and RF), ECG measurements	Not reported	AUROC 0.83 (RF model)	Sensitivity 63.2%, specificity 94.6% (RF model)
Tsuji et al., (2020) [28]	Single-center, retrospective observational study	A dataset from Physionet	20 patients (20 patients with cardiac arrest)	30 s single-lead ECG recordings, HRV	VF prediction within 1 min (up to 7 min)	RNN (HRV)	Not reported	AUROC 0.94 (1 min to VF)	Accuracy 90.0% (1 min to VF)
Kwon et al., (2020) [18]	Multi-center (*n* = 2), retrospective observational study	Admitted patients (two hospitals)	24,600 patients and 47,000 ECGs (1054 patients and 2298 ECGs with cardiac arrest)	10 s 12-lead ECGs ECG waveform	Cardiac arrest prediction within 24 h	CNN (ECG waveform)	Internal + external validation	AUROC 0.91 (internal validation), AUROC 0.95 (external validation)	Sensitivity 88.3%, specificity 83.8%, PPV 8.7%, NPV 99.8% (internal validation)
Lee et al., (2023) [21]	Single-center, retrospective observational study	Hospitalized patients, intensive care unit	4821 patients (107 patients with cardiac arrest)	Serial 5 min single-lead ECG recordings, HRV	Cardiac arrest prediction within 24 h	DT-based algorithm (HRV)	Internal validation	AUROC 0.88, AUPRC 0.11	Sensitivity 81.7%, specificity 80.0%, precision 5.3%, accuracy 80.9%, F1 score 10.0%
Oberdier et al., (2025) [29]	Single-center, retrospective observational study	A publicly available dataset (Nightingale Open Science-Subtyping Cardiac Arrest dataset)	1326 patients (221 patients with cardiac arrest)	10 s 12-lead ECGs (segments focused on the R wave), ECG waveform	Cardiac arrest prediction within 24 h (secondary analyses up to 1 year)	CNN (ECG waveform + clinical characteristics)	Internal validation	AUROC 0.77	Sensitivity 95.0%, specificity 31.1%, accuracy 84.8%, PPV 87.8%, NPV 52.2%, F1 score 91.3%
Fiorina et al., (2025) [26]	Single-center, retrospective observational study	Publicly available international ambulatory dataset	250,000 recordings (1104 recordings with VT)	14-day single-lead ambulatory ECG recordings (24 h input), ECG waveform	Sustained VT prediction within 13 days	CNN (ECG waveform + heart rate data)	Internal + external validation	AUROC 0.96, AUPRC 0.30 (internal validation), AUROC 0.95 (external validation)	Sensitivity 70.6%, specificity 97.7%, PPV 12.3%, NPV 99.9% (internal validation)

Short-term risk stratification studies: ECG—electrocardiogram, AI—artificial intelligence, HRV—heart rate variability, SVM—support vector machine, AUROC—area under a receiver operating characteristic curve, PPV—positive predictive value, NPV—negative predictive value, VT—ventricular tachycardia, ML—machine learning, SCD—sudden cardiac death, KNN—K-nearest neighbors algorithm, DT—decision tree-based algorithm, RF—random forest model, VF—ventricular fibrillation, RNN—recurrent neural network, CNN—convolutional neural network, AUPRC—area under precision-recall curve.

**Table 2:** Long-term risk stratification studies.

Study (Year of Publication)	Study Type	Population and Setting	Number of Patients/Recordings	ECG Modality and Analyzed Data	Outcome	AI Model Type and Included Parameters	Validation	Model Discrimination	Other Performance Metrics
Rodriguez et al. (2019) [30]	Single-center, retrospective observational study	DCM cohort	140 patients (77 patients with SCD/cardiac arrest)	30 min single-lead ECG recordings, HRV	SCD or cardiac arrest prediction within a mean follow-up period of 28 months	SVM (HRV + blood pressure measurements)	Not reported	AUROC 0.94	Sensitivity 93.7%, specificity 95.5%, accuracy 93.6%
Sammani et al. (2022) [31]	Multi-center (*n* = 2), retrospective observational study	DCM cohort (two hospitals)	695 patients (115 with cardiac arrest or sustained VT)	10 s 12-lead ECGs, ECG waveform	SCD or sustained VT/VF or appropriate ICD therapy prediction within a median follow-up of 51 months	An explainable DNN (ECG waveform-based 21 generative factors—e.g., T wave height and orientation)	Internal validation	AUROC 0.67	Not reported
Kolk et al. (2023) [19]	Multi-center (*n* = 2), retrospective observational study	ICD cohort (two hospitals)	2942 patients and 32,129 recordings (840 patients with a VT/VF)	Serial 10 s 6-lead ECGs, longitudinal ECG waveform data	Sustained VT/VF prediction within 3 months (a median follow-up of 44 months)	A dynamic ML model (longitudinal ECG data + clinical characteristics) + DL model (ECG waveform)	Internal validation	AUROC 0.74 (dynamic model), AUROC 0.64 (static model)	Not reported (+model calibration plots)
Shiraishi et al. (2023) [33]	Multi-center (*n* = 4), prospective observational study	Heart failure cohort (dataset from four hospitals)	2559 patients (48/1077 SCD patients in the testing cohort)	10 s 12-lead ECGs, ECG waveform	SCD or appropriate ICD therapy prediction within 36 months	RNN + CNN (ECG waveform, clinical characteristics (NYHA class and EF < 35%))	Internal + external validation	AUROC 0.66 (ECG-AI + clinical model), AUROC 0.62 (ECG-AI)	Sensitivity 0.635, specificity 0.648, Hosmer–Lemeshow test *p* = 0.11 (ECG-AI + clinical model)
Nakamura et al. (2023) [37]	Single-center, retrospective observational study	Brugada syndrome cohort	157 patients and 2053 recordings (16 patients with VF and (549 recordings))	10 s 12-lead ECGs, ECG waveform	VF prediction within a median follow-up of 42 months	CNN (ECG waveform)	Internal validation	AUROC 0.80	Accuracy 77.1%, PPV 44.2%, NPV 94.1%, precision 93.2%, F1 score 81.1%
Holmstrom et al. (2024) [20]	Multi-center (*n* = 2), retrospective observational study	Community SCD cohorts, (Oregon SUDS + Ventura PRESTO)	3835 patients and 3900 recordings (2510 patients with SCD)	10 s 12-lead ECGs, ECG waveform	SCD prediction within a median follow-up of 20 months	CNN (ECG waveform, clinical variables)	Internal validation + external validation	AUROC 0.89 (ECG-AI, internal validation), AUROC 0.82 (ECG-AI, external validation), AUROC 0.92 (ECG-AI + clinical variables)	Sensitivity 88.9%, specificity 84.3%, F1 score 86.6% (ECG-AI internal validation)
van de Leur et al. (2024) [32]	Multi-center (*n* = 3), retrospective observational study	Phospholamban cardiomyopathy cohort (nationwide registry)	679 patients (72 patients with cardiac arrest or sustained VT)	10 s 12-lead ECGs, ECG waveform	SCD or sustained VT or appropriate ICD intervention prediction within 60 months.	An explainable DNN (ECG waveform-based 21 generative factors)	Internal validation	AUROC 0.86, AUPRC 0.27	Sensitivity 92.0%, specificity 52.0%, PPV 11.0%, NPV 99.0%
Baker et al. (2024) [25]	Multi-center (*n* = 2), retrospective, observational study	Ambulatory dataset (two hospitals)	270 patients (159 patients with sustained VT/VF)	24 h 3-lead ambulatory ECG recordings (10 s input), ECG waveform	Sustained VT/VF prediction within a median follow-up of 19 months	CNN (ECG waveform)	Internal validation	AUROC 0.80	Sensitivity 78.0%, specificity 73.0%, PPV 81.0%, NPV 70,0%, accuracy 76.0%, F1 score 79.0%
Kolk et al. (2024) [34]	Multi-center (*n* = 2), retrospective observational study	NICM ICD cohort (two hospitals)	289 patients (26 patients with a sustained VT/VF)	10 s 12-lead ECGs, ECG waveform	Sustained VT/VF or appropriate ICD therapy prediction within a mean follow-up of 12 months	Multimodal ML model (LGE-MRI scans, ECGs, clinical characteristics) + DL model (ECG waveform)	Internal validation + external validation	AUROC 0.84, AUPRC 0.31 (multimodal model), AUROC 0.64 (ECG-AI only)	Sensitivity 98.1%, specificity 72.6%, accuracy 74.1% (+model calibration plots)
Järvensivu-Koivunen et al. (2024) [38]	Single-center, retrospective observational study	Post-ACS cohort (MADDEC database)	8568 patients, (287 patients with SCD)	10 s 12-lead ECGs, QRS axis, amplitude and duration, QTc interval, ST segment, T wave axis, PVC	SCD or appropriate ICD therapy prediction within a median follow-up 5 years	Conventional ML algorithms (logistic regression, XGB, RF), ECG measurements	Internal validation	AUROC 0.693 (XGB), AUROC 0.681 (RF model)	Sensitivity 9.0%, specificity 98.1%, PPV 13.1%, NPV 97.5% (XGB)
Tateishi et al. (2025) [35]	Single-center, retrospective observational study	ICD cohort	200 patients (59 patients with appropriate ICD therapy)	10 s 12-lead ECG, RR interval, QTc interval, ST segment	Appropriate ICD therapy prediction within a median follow-up of 98 months	Unsupervised ML algorithms (K-means and hierarchical clustering)	Not reported	Not reported	Silhouette coefficient = 0.6, Cluster 1 HR = 1.53

Long-term risk stratification studies: ECG—electrocardiogram, AI—artificial intelligence, DCM—dilatative cardiomyopathy, SCD—sudden cardiac death, HRV—heart rate variability, SVM—support vector machine, AUROC—area under a receiver operating characteristic curve, VT—ventricular tachycardia, VF—ventricular fibrillation, ICD—implantable cardioverter–defibrillator, DNN—deep neural network, ML—machine learning, DL—deep learning, RNN—recurrent neural network, CNN—convolutional neural network, NYHA—New York Heart Association, EF—ejection fraction, PPV—positive predictive value, NPV—negative predictive value, AUPRC—area under precision-recall curve, NICM—non-ischemic cardiomyopathy, LGE—late gadolinium enhancement, MRI—magnetic resonance imaging, ACS—acute coronary syndrome, PVC—premature ventricular contraction, XGB—extreme gradient boosting model, RF—random forest model, HR—hazard ratio.

## Data Availability

No new data were created or analyzed in this study. Data sharing is not applicable to this article.

## Supplementary Materials

The following supporting information can be downloaded at: https://www.mdpi.com/article/10.3390/jcdd13050206/s1. Table S1—PROBAST-based risk of bias assessment and applicability concerns: PROBAST—Prediction model Risk of Bias Assessment Tool.
