# Supplementary material for: Artificial Intelligence-Enabled Electrocardiography for Prediction of Sudden Cardiac Death and Malignant Ventricular Arrhythmias: A Scoping Review
Source: J Cardiovasc Dev Dis. 2026 May 12;13(5):206. doi: 10.3390/jcdd13050206 (PMC13207818; doi:10.3390/jcdd13050206)
Supplement: Supplementary file 1 [file jcdd-13-00206-s001.zip › jcdd-4257168-supplementary.pdf]

TABLE S1 - PROBAST-based Risk-of-Bias Assessment and Applicability concerns

Study (year of publication)	Risk-of-bias assessment					Applicability concerns		
	Population	Predictors	Outcome	Analysis	Overall	Population	Predictors	Outcome
Ong et al, (2012) [24]	Low	Low	Low	High	High	Low	Low	Low
Lee et al, (2016) [36]	High	Unclear	Low	High	High	Unclear	Low	Low
Lai et al, (2019) [27]	High	Unclear	Unclear	High	High	High	Low	Unclear
Rodriguez et al, (2019) [30]	Low	Low	Low	High	High	Low	Low	Low
Do et al, (2019) [17]	Unclear	Low	Low	High	High	Low	Low	Low
Tsuji et al, (2020) [28]	High	Low	Low	High	High	Low	High	Low
Kwon et al, (2020) [18]	Low	Low	Low	Unclear	Unclear	Low	Low	Low
Sammani et al, (2022) [31]	Unclear	Low	Low	Unclear	Unclear	Low	Low	Low
Lee et al, (2023) [21]	Unclear	Low	Low	High	High	Low	Low	Low
Kolk et al, (2023) [19]	Low	Low	Low	Low	Low	Low	Low	Low
Shiraishi et al, (2023) [33]	Low	Low	Low	Low	Low	Low	Low	Low
Nakamura et al, (2023) [37]	High	Low	Low	High	High	Low	Unclear	High
Holmstrom et al, (2024) [20]	Low	Low	Low	Low	Low	Low	Low	Low
Van der Leur et al, (2024) [32]	Unclear	Low	Low	High	High	Low	Low	Low

Study (year of publication)	Risk-of-bias assessment					Applicability concerns		
	Population	Predictors	Outcome	Analysis	Overall	Population	Predictors	Outcome
Barker et al, (2024) [25]	High	Low	Unclear	High	High	Low	Unclear	High
Kolk et al, (2024) [34]	Low	Low	Low	Unclear	Unclear	Low	Low	Low
Järvensivu-Koivunen et al, (2024) [38]	High	Low	Low	High	High	Low	Low	Low
Oberdier et al, (2025) [29]	Unclear	Low	Low	High	High	Low	Low	Low
Fiorina et al, (2025) [26]	Low	Low	Low	Low	Low	Low	Low	Low
Tateishi et al, (2025) [35]	Unclear	Low	Low	High	High	Low	Low	Low
